# A Retrospective Study (2015–2020) on the Risk Factors Associated with the Persistence and Spread of Brucellosis in Buffalo Farms in Caserta Province, Italy

**DOI:** 10.3390/vetsci11030119

**Published:** 2024-03-06

**Authors:** Maria Ottaiano, Roberta Brunetti, Antonio Limone, Maria Rosaria Capone, Alessandra Di Giuseppe, Annamaria Conte, Fabrizio De Massis, Paolo Chiodini, Simona Signoriello, Loredana Baldi, E. De Carlo

**Affiliations:** 1 Experimental Zooprophylactic Institute of Southern Italy, 80055 Portici, Italy; maria.ottaiano@izsmportici.it (M.O.); antolim@izsmportici.it (A.L.); loredana.baldi@izsmportici.it (L.B.); 2 Reference Laboratory for Brucellosis (Brucella abortus, Brucella melitensis, Brucella suis), Reference Laboratory for Ovine Epididymitis (Brucella ovis), Experimental Zooprophylactic Institute of Abruzzo and Molise “G. Corporal, Via Campo Boario, 1, 64100 Teramo, Italy; m.capone@izs.it (M.R.C.); a.conte@izs.it (A.C.); f.demassis@izs.it (F.D.M.); 3 Department of Mental and Physical Health, Preventive Medicine University of Campania “Luigi Vanvitelli” Largo Madonna Delle Grazie, 1, 80138 Naples, Italy; paolo.chiodini@unicampania.it (P.C.); simona.signoriello@unicampania.it (S.S.); 4 National Reference Centre for Hygiene, Technologies of Water Buffalo Farming and Productions-Experimental Zooprophylactic Institute of Southern Italy, 80055 Portici, Italy; esterina.decarlo@izsmportici.it

**Keywords:** brucellosis, risk factors, regression model, statistical association, buffalo, Caserta

## Abstract

**Simple Summary:**

This work addressed the age-old problem of buffalo brucellosis in the province of Caserta; this infection has repercussions both on public health (disease transmissible to humans) and on the economy of the DOP (Protected Designation of Origin) buffalo mozzarella dairy supply chain. This disease has persisted for years in the Caserta area, and numerous resources have been invested but to no avail. The aim of the work was to identify the main causes of the persistence of the contagion in order to act on them and improve the use of public funds. The study showed that the infection persists in the same companies and that it has most likely never moved away from them and is always present in the same areas where the companies are close to each other, and the disease has spread from one company to another due to the absence of disease surveillance and quarantine measures. In fact, companies that have had a previous history of infection and/or are close to other infected companies have a greater probability of contracting the infection.

**Abstract:**

Bovine and bubaline brucellosis is still present in some regions of Italy. Although control and eradication measures have been implemented for several years, the brucellosis situation remains problematic in the Campania region. The infection is present in the provinces of Salerno and Caserta, with the latter experiencing a drastic increase in the prevalence and incidence of infection in buffalo species (Bubalus bubalis) in recent years. The brucellosis eradication plan in Italy is subject to the European co-financing system, and failure to achieve the objectives of the plan has resulted in economic cuts for the Campania Region for years. This study aimed to evaluate the possible risk factors associated with the spread and persistence of brucellosis infection on buffalo farms in the Province of Caserta. The results of official controls carried out from 2015 to 2020 on the buffalo farms of the Province were analyzed. Statistical analysis was performed by means of the R software (version 4.1.0) on a final dataset consisting of 4583 observations. The possible association between covariates and outcome (presence/absence of infection) was evaluated (T-Fisher and Wilcoxon). A logistic regression model with mixed effects was carried out. The study shows that the risk of infection is statistically associated with the density of farms per square km and previous notifications of abortions on the same farms. Furthermore, animal movements constitute a risk factor for the permanence of infection over time (OR > 1), and herds already infected prior to 2015 were seen to have an almost three-fold higher risk of developing the disease (OR = 3.35).

## 1. Introduction

Brucellosis is a zoonosis caused by a Gram-negative bacterium of the genus Brucella which affects humans and animals it is considered one of the most widespread zoonotic infections worldwide and can undoubtedly be considered, especially in areas where it becomes endemic, an “occupational” disease, as it mainly affects breeders, veterinarians, slaughterhouse employees, and butchers [1,2,3,4,5,6,7,8,9].

On average, about 500,000 cases (Noah C et al., 2018 [10]) of human brucellosis are reported annually, but estimates from the World Health Organization (WHO World Health Organization, 2006 [11]) suggest that, owing to underreporting in disease surveillance activities, the true incidence could be 10 to 25 times greater (Shirima GM—Tanz J Hlth Res (2010) [12]).

Historically, the European countries most affected by brucellosis are those in the Mediterranean area. The data collected in 2008 reveal that about 85% of reported cases of human brucellosis occurred in Greece, Italy, Portugal, and Spain. Furthermore, it has also been ascertained that almost all of the cases reported in Northern European countries are “imported cases”, i.e., involving travelers returning from countries where brucellosis is endemic [10,13].

It should be emphasized that although it is considered an occupational infection, especially in endemic areas, the disease does not only affect professionals; it is also a real risk for the general population, which can be infected through the consumption of fresh dairy products obtained from raw milk (EFSA, 2010–EFSA 2022, [13]).

Although this zoonosis has been eradicated in some countries, it is still present in many areas of Europe and causes considerable damage in various techno–commercial sectors, with economic losses being due to late abortions and/or stillbirths, the culling of infected animals and the destruction or heat treatment of milk and meat products.

The infection of a herd causes economic losses due to abortions (which occur between the 3rd and 4th months of pregnancy, with possible retention of the fetal membranes) and decreased milk production (Calistri et al., 2013 [14]). Most infected cows abort only once in their lifetime, although the placenta may still be infected during subsequent gestations. Brucella localizes in the supramammary lymph nodes and mammary glands in 80% of infected animals, which shed the pathogen in their milk for life [15,16].

An important risk factor for the spread of the infection is promiscuity, i.e., the contact of animals from different farms; sharing the same open pastures for long periods facilitates the transmission of the infection from infected farms to healthy farms through environmental contamination due to aborted fetuses, placentas, lochia, exudates, and uterovaginal secretions from infected animals (Olsen and Tatum, 2010 [17]). Factors favoring the persistence of the bacterium are lack of an adequate surveillance system, high density of animals, close contact between different susceptible species, and poor management and low level of herd biosecurity [18,19]. The main risk factors include the introduction of an infected animal into a healthy population; incorrectly managed abortions; the use of contaminated milk, drinking water, or food; and poor veterinary practices (use of contaminated tools) [20,21].

Infected animals shed B. abortus through colostrum and milk after delivery; this excretion can continue intermittently for many months and, in some cases, even for more than two years. The udder of an infected animal is the organ most frequently colonized by Brucella. Moreover, urine and feces contribute to contamination of the environment, with consequent indirect transmission of the infection to susceptible animals (Centers for Disease Control and Prevention CDC) https://www.cdc.gov/brucellosis/index.html—1 March 2022 [22]).

In Italy, an eradication strategy was introduced in 1994 (Ministry of Health, 1994 [23]) when a policy of vaccination, stringent testing, and slaughter was adopted. The plan aimed to eradicate brucellosis from cattle farms in five years (Calistri et al., 2013 [14]). However, despite the application of control and eradication measures for several years, brucellosis remains a problem in some southern regions of Italy [14,24]. Italy is currently divided quite clearly, with the regions of Northern Italy free from infection for years, while in some regions of Central and especially Southern Italy, brucellosis persists with a particularly high prevalence in some provinces (https://www.salute.gov.it/portale/temi/p2_4.jsp?lingua=italiano&area=sanitaAnimale—1 March 2022) [25].

The national eradication plan is based on the periodic serological testing of cattle (Bos Taurus) and buffalo (Bubalus bubalis) herds. The interval between tests and the number of herds and animals to be tested vary according to the health status of the province concerned. Since 2015, eradication measures have been intensified, and stricter provisions have been issued for the detection and slaughter of infected animals (Ministerial Ordinance May 2015: Extraordinary veterinary police measures regarding tuberculosis, bovine and buffalo brucellosis, sheep and goat brucellosis, enzootic bovine leucosis [21,26]).

In the Campania Region, the provinces of Naples, Avellino, and Benevento were declared free from bovine brucellosis in 2021, and the obligation to vaccinate was lifted (Decision 385 of the EU of March 2021, https://eur-lex.europa.eu/legal-content/EN/TXT/?uri=CELEX:32021D0385—1 March 2022 [27]). In Salerno Province, although the disease is still present, the trend in the incidence of infections has progressively declined over the last three years (Refai, M et al., 2002 [3]). In Caserta Province, however, buffalo brucellosis is still a serious problem, both because of its possible repercussions on human health and because of the huge economic and commercial losses that it causes in the zootechnical sector.

Campania is the region with the highest concentration of buffalo herds in Italy, and about 80% of Campania’s buffalo population is localized in the territory of the Province of Caserta (https://www.vetinfo.it/ (accessed on 1 March 2022) [28]). This zootechnical activity, which involves over 1400 farms and about 300,000 animals, occupies a prominent position in the overall agricultural system and is an important component of the region’s economy. An average of 50,000 tons of “Campania buffalo mozzarella cheese DOP” (protected denomination of origin) is produced per year, accounting for about €430 million (34% is exported), with the overall value of the supply chain reaching €1.5 billion (about 1.5% of regional GDP). However, the brucellosis eradication plan in Italy is subject to the European co-financing system, and failure to achieve the objectives of the plan has penalized the Campania Region economically for years.

In the last six years, a drastic increase in infections in the Caserta area has been observed (https://www.vetinfo.it/ (accessed on 1 March 2022) [28]). Every year, numerous new outbreaks have been detected, testifying to the failure of the prevention and control system and suggesting the need for an in-depth assessment of the current epidemiological situation; this means identifying the main risk factors for the persistence and spread of bovine brucellosis in the buffalo herds of the Caserta Province.

The aim of this study was to evaluate the possible risk factors that influenced the spread and persistence of brucellosis infection in Caserta buffalo herds in the period 2015–2021 in order to plan appropriate countermeasures. These presuppose the efficient and effective use of the available resources and the provision of practical support for the local veterinary services in evaluating and planning diagnostic, preventive, and control interventions.

## 2. Materials and Methods

A retrospective longitudinal cohort study was carried out on data from buffalo herds in Caserta Province over a 6-year period (from 2015 to 2020). These data were extracted from the National Veterinary Information Systems (https://www.vetinfo.it/ (accessed on 1 March 2022) [28]) and from the Laboratory Management System (SIGLA) of the Experimental Zooprophylactic Institute of Southern Italy.

Data were retrieved from the following national databases:

- SANAN: Animal Health Information System; this web application for Animal Health is accessible from the Portal of the Veterinary Information System, to which the territorial veterinary service uploads the results of health examinations carried out on each farm. Through SANAN, the local health units and the Ministry of Health can monitor the progress of eradication activities and carry out specific risk analyses;
- SIMAN: the centralized notification system for infectious disease outbreaks in animals; it collects all information on notifiable disease outbreaks;
  - NDb: the national database of farms and animals, in which all herds and bovine animals are recorded, including all movements of each animal throughout its life.

The data provided by SIGLA were used to collect the outcomes (positive/negative) of serological testing and the presence of abortions in each herd. The data provided by NDb were used to identify the herd’s geolocation, the presence of other animals in the herd (cattle and/or sheep and goats), the number of movements from or to the herd, the number of animals, and the herd’s health status. SIMAN provided information on confirmed outbreaks of bovine brucellosis before 2015.

The results of official controls carried out from 2015 to 2020 on the buffalo farms of the Caserta Province were analyzed. Farms were classified as infected (positive) even when only one animal proved positive on serological testing. According to European provisions, all bovine animals must be serologically tested twice a year. The Rose Bengal test (RBT) is used as a screening test. All RBT-positive animals are also tested by means of the complement fixation test (CFT). Positive animals are considered infected and are slaughtered.

The following inclusion criteria were used to select the herds to be studied.

- Inclusion criteria:
  ▪ presence of buffaloes in the herd in the period 2015–2020;
  ▪ herds located in Caserta Province;
  ▪ herds controlled for brucellosis at least once in the years 2015–2020.
- Exclusion criteria:
  ▪ herds never checked for Brucellosis in the period considered;
  ▪ herds located outside the province of Caserta;
  ▪ herds with animals less than 12 months old;
  ▪ herds not subject to the Brucellosis program.

Statistical analysis was carried out by means of the R software version 4.1.0. (R Foundation for Statistical Computing, Vienna, Austria). Continuous variables were reported as average, standard deviation, median, minimum, and maximum values, whereas categorical variables were reported as percentages (Ricci, V., 2006 [29]).

A preliminary analysis was conducted on positive farms by calculating the percentage of recurrent positive results throughout the entire period considered: 231 farms were infected, 39% of which had previously proved positive. As this percentage was less than 50%, the statistical analysis was able to be carried out. If the percentage of farms with recurrences of confirmed outbreaks had exceeded 50%, many of the observations would have been on the same positive farms, and it would, therefore, not have been possible to identify the risk factors.

The analyses were carried out for each year, and the possible dependence of the outcome on each single covariate was evaluated. The Fisher’s exact test was used for qualitative variables, while the non-parametric Wilcoxon test was used for quantitative variables. The results were considered significant if the *p*-value was <0.05.

The variables analyzed are listed below:

- Presence of an outbreak three years earlier: dichotomous variable Yes/No, which indicates whether an outbreak of bovine brucellosis had been recorded in the herd three years before the positivity found in the year of concern.
- Presence of an outbreak in the previous year: dichotomous variable Yes/No, which indicates whether an outbreak had been recorded in the herd in the year before the positivity found in the year of concern.
- Withdrawal of free status: dichotomous variable Yes/No, indicating whether the herd had lost its brucellosis-free status in the previous years.
- Presence of sheep and goats: dichotomous variable Yes/No, indicating whether sheep and goats are also kept in the same herd.
- Density of farms per square kilometer, calculated according to the kernel function, which takes into account the distance between farms (the density value is higher in areas close to a farm and decreases as the distance from it increases).
- Density of animals per square kilometer, calculated according to the kernel function, which takes into account the distance between farms. The number of animals in the herd determines the number of times that the herd is considered in the analysis (for example, a herd with 3 animals is considered as if there were 3 herds in the same area.
- Movements: dichotomous variable Yes/No, indicating whether the herd introduced animals during the year of concern. Specifically, for positive farms, only introductions during the 6 months before the first positive result were considered, while for negative farms, only introductions during the 6 months before the last serological control carried out in the year of concern were considered.
- Number of movements: a numerical variable that indicates the number of movements carried out according to the criteria described above.
- Number of animals moved: a numerical variable indicating the number of animals moved according to the criteria described above.
- Presence of positivity in the herd before the period under study: dichotomous variable Yes/No, which indicates whether the herd had suffered a confirmed outbreak in the period before the years under investigation.
- Abortions: dichotomous variable Yes/No, indicating whether abortions occurred in the herd during the period under study.

Mixed effects logistic regression is used to model binary outcome variables, the logistic probabilities of outcomes being modeled as a linear combination of the predictor variables when the data are pooled or when there are both fixed and random effects. A mixed model is a statistical model containing both fixed effects and random effects. The response variable is then modeled by combining fixed effects, which are common to the whole population, with random effects, which vary among individuals.

Mixed-effects logistic regression was performed, as the dependent variable is a dichotomous variable, and there are multiple predictors.

Since the number of movements, number of animals moved, and movements were correlated, we decided to include only the “movements” variable in the model as an explanatory variable, as this was statistically associated with the result during the preliminary analysis.

Before being included in the model, the Yes/No dichotomous variables were transformed into “factors” by associating them with the presence (“1”) or absence (“0”) of the covariate.

A mixed-effects logistic regression model was then constructed in order to evaluate the effect of possible factors over time and quantify any risk for the development of the disease; the model included only the explanatory variables that displayed a significant association with the response variable and those covariates which were significant in the univariate analysis with the iteration with the time variable. In the model, the response variable was constituted by the presence or absence of disease (dichotomous variable), the fixed effects were represented by the temporal variable (year), and the random effects were represented by the individual farms.

The significance of the variables within the final model was confirmed by means of the ANOVA test (significance for *p*-value < 0.05).

After the construction and results of the model, the odds ratio (OR) was calculated as the exponential of the regression coefficients obtained.

We carried out a spatial autocorrelation analysis by using the prevalence values at the municipality level; this analysis was performed to support the significance of the covariate “density of farms per square km” (Kernel) in the model. The analysis was run in two steps: the first one was to evaluate the global autocorrelation through the calculation of Moran’s Index, and the second one was to identify possible outliers at the local level by means of the Anselin Local Moran’s I.

## 3. Results

The dataset consisted of 4583 observations and nine variables regarding 861 herds. During the entire period considered, 231 farms were infected, 39% of which suffered repeated outbreaks of bovine brucellosis.

The analysis for each year revealed a statistically significant association between the presence of the disease on the farm and the following qualitative variables: reported abortions, withdrawal of the brucellosis-free health status, and number of movements (from Table 1, Table 2, Table 3, Table 4, Table 5 and Table 6).

Furthermore, for the variables “density of farms per square km” and “density of animals per square km”, a statistically significant difference was found between infected herds and those that always tested negative in the period.

Given that many herds were repeatedly positive over the years, an analysis was carried out in which the overall period was considered. The following variables proved to be significantly associated with the positive status of the herd in the period: presence of abortions, presence of sheep and goats, positive status of the herd before 2015, the number of animals in the herd, and the density of herds and animals per square kilometer.

More than 50% of the positive herds in the period considered had suffered an outbreak of bovine brucellosis in the period prior to the one examined (2008–2014); a statistically significant association was found between the presence of the infection and a positive status of the herd in the previous years, confirming a tendency of the infection to persist in the same herds.

In addition, the infection was more frequently present on farms with a greater number of animals. This is not surprising, given the epidemiology of the disease; a greater number of animals means a higher probability of shedding Brucella via abortion or birth (Calistri et al., 2013 [14]).

The number of movements carried out, and the number of animals introduced from other farms do not seem to play important roles in the persistence of brucellosis. Furthermore, a statistically significant difference was found for the variable “average number of animals in the herd” in the two groups of the outcome.

Herds that had their brucellosis-free status withdrawn had a 3.5 times higher risk of testing positive than herds with a history of maintenance of their infection-free status. Similarly, those with notifications of abortions had an approximately 2.5-fold higher probability of being positive (Table 7). Movements constituted a risk factor for the development of the disease over time, albeit lower than other features. As can be seen from the table above, the OR was higher than unity, albeit slightly. In 2019, the herds into which animals had been introduced in the previous period displayed a 15% higher probability of developing the disease than those that did not introduce animals.

**Figure 1:**
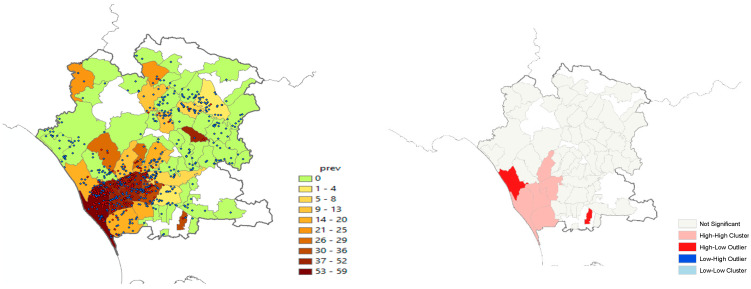
Spatial’s Autocorrelation.

Furthermore, it can be seen that the risk of infection was three times greater where the density of farms per km was higher. On average, the global Moran’s Index was statistically significant (Moran’s index = 0.14, Z-value = 2.03, *p* < 0.05), showing a clustering of the infection, expressed as the prevalence of positive herds in each municipality. The local analysis identified a few hot-spot municipalities: two High–Low (red color) and five High–High (pink color) (Figure 1). The intensity of prevalence proved similar in neighboring areas, i.e., it is more likely that neighboring municipalities have similar prevalence values and that they influence each other. The municipalities in red represent outliers or anomalous values, as they displayed higher prevalence values than those found in neighboring areas.

Subsequently, a univariate model was constructed for the variable “pre-2015 positivity”; herds that had suffered an outbreak prior to 2015 were found to have an almost three-fold higher risk of developing the disease (Table 8).

## 4. Discussion

The data used in this study are collected on a regular basis, the main purpose being to monitor the activities and results of the eradication plan. The use of data collected ad hoc would have allowed a broader and more specific vision but would have proved costly. Furthermore, for obvious economic reasons, an ad hoc study cannot be carried out as a census of the entire animal population of a region; therefore, the representativeness of the data collected is greater than that provided by an ad hoc data study (Calistri et al., 2013 [14]). The system of recording all control activities has the purpose of monitoring the progress of the plan and the achievement of its objectives. Also, the availability of this large amount of data is essential in order to carry out adequate epidemiological analyses, which are fundamental to the correct rescheduling of activities based on risk analysis. Indeed, in the final phase of an eradication program, it is necessary to identify possible risk factors that affect the spread and maintenance of infection in the animal population and to search for any residual sources of infection (Nannini et al.,1992 [24]). From the analyses carried out, it can be seen that the contagion persists in the same areas with a resurgence in recent years, recurring cyclically in the same farms. The significant association between the presence of the disease in the previous years of the period considered and the outcome of the study (presence of the disease) can undoubtedly be considered as further evidence of the persistence of the infection in the same herds and denotes an evident difficulty of eradication in some animal subpopulations. Historically, infected herds are more likely to become infected again over time. The identification of so-called “problematic” farms, i.e., those in which the infection has recurred for several years, becomes of crucial importance for their correct management (Calistri et al., 2013 [14]). Given the recurrence of the disease in the same farms, it is plausible that it has never been eliminated from the farm itself or from the group of contiguous farms forming part of the same epidemiological unit. This information allows us to focus attention on the critical issues that influence the achievement of the set objective, i.e., that of obtaining the health status of “free” from infection, both at a territorial and company level. In the province of Caserta, nearby farms often lack effective separation of the herds, both physically and managerially; this is confirmed by the statistically significant association between the density of farms per square kilometer and their infectious status; indeed, the risk of contracting the infection increases considerably as the density of farms per square kilometer increases. In this regard, the concept of “biosafety” is of fundamental importance: introduced by the new EU Regulation on Animal Health 429/2016, this concept constitutes one of the main means to avoid the introduction, development, and spread of transmissible animal diseases within an animal population and in the surrounding environment [30,31]. Furthermore, the implementation of biosecurity measures should take into account both the local context in which the farms are located and the epidemiological situation of the area. Inadequate farm management can favor the circulation of the disease in the herd through contact with bacteria from young animals, in which the infection is detected only after months (puberty period). Bear in mind that an animal that becomes infected can reveal itself to be positive even after 6 months. Added to this is the fact that enormous quantities of Brucella are released into the environment with the products of childbirth and abortions (Benkirane, A. et al., 2015 [32]). The impossibility of interrupting both calving and milking during an epidemic constitutes a major problem to which there is no solution other than the perfect management and structuring of farms according to correct biosecurity criteria. Slaughtering all pregnant animals, even negative ones, is unthinkable. With our study, we found that in the province of Caserta, the main cause of the persistence of the infection in some areas is attributable exclusively to the proximity of buffalo farms. It is likely that some farm owners directly or indirectly (wives/children) own other farms, and, in such cases, it is crucial to extend restrictive measures in order to mitigate the risk of disease spread in the event of a suspected outbreak and/or confirmed. Although animal movements appear to have played a marginal role in the spread of the infection, in the years following 2017, they appear to have had a greater impact. This is probably attributable to the failure to meet deadlines for re-checking farms in 2017. Our data also reveals that timely notification of abortions to the Competent Authority is of crucial importance for the containment of contagion; it is necessary to highlight that “pathological” material is often sent by individual operators in the sector to the official laboratory for the search for possible pathogenic agents, without prior communication to the veterinary services of the competent authority. This compromises the effectiveness of risk mitigation measures in the phase of suspicion of infection on the farm. The present study also confirms that herds that have had their brucellosis-free status suspended are more likely to develop the disease. This finding should lead the Competent Authority, as soon as the presence of infection in the herd is suspected, to implement management measures aimed at mitigating the possible risk of spreading the pathogenic agent to the entire herd and to evaluate all the elements in its possession in order to carry out correct and well-founded epidemiological reasoning aimed at confirming or not the infection. In light of the conclusions reached, it is necessary for the Competent Authorities to modify their control programs, taking into account the method according to which all companies, even if equipped with different codes, are contiguous, are considered to be part of a single epidemiological unit, within which all individual companies must therefore be managed and controlled simultaneously and be subjected to any preventive and/or restrictive measures. Finally, the involvement of all those involved, especially stakeholders, is essential to increase awareness of one’s responsibilities. In fact, without the will of farmers to definitively solve the problem, any measures adopted will not be effective. Cooperation and sharing are necessary to achieve the objective of progressively reducing the problem of brucellosis, thus safeguarding not only the Caserta livestock heritage but an entire economic sector.

## 5. Conclusions

Brucellosis in buffaloes in Caserta is still a serious problem that impacts the entire Region of Campania. Over the years, numerous funds have been allocated for the eradication of this infection, albeit with poor results. Our study focused on critical issues and risk factors responsible for the persistence and resurgence of buffalo brucellosis in the province of Caserta. Unlike what has been found in sheep and goats, where the disease appears to have spread mainly due to the movement of animals, with regard to buffaloes, the disease has mostly spread by passing from one farm to another due to the concentration of the herds themselves in certain areas [33,34]. On the other hand, this result is perfectly consistent with the different breeding methods of the two species; buffalo farms are mostly intensive and concentrated farms adjacent to each other, and this has led to direct contact between animals coming from different farms, even in the absence of movements tracked in information systems. This analysis provides tools that can help you focus your resources where necessary without waste. The fact that farms that previously had their own suspended qualifications presented a higher risk of infection highlights the need for improvement in the management of livestock farms, especially in the case of large companies. To date, this aspect has been underestimated. Our study provides concrete evidence of what has already happened and has been reported in the literature.

## Figures and Tables

**Table 1:** 2015 Fisher test and Wilcoxon test.

	Negative	Positive	*p*-Value
	(N = 791)	(N = 26)	
Presence of cattle on the farm			
No	464 (58.7%)	16 (61.5%)	0.928
Yes	327 (41.3%)	10 (38.5%)	
Presence of sheep and goats on the farm			
No	763 (96.5%)	26 (100%)	0.668
Yes	28 (3.5%)	0 (0%)	
Movements			
No	617 (78.0%)	23 (88.5%)	0.302
Yes	174 (22.0%)	3 (11.5%)	
Abortions			
No	775 (98.0%)	24 (92.3%)	0.208
Yes	16 (2.0%)	2 (7.7%)	
Positive three years earlier			
No	771 (97.5%)	19 (73.1%)	<0.05
Yes	20 (2.5%)	7 (26.9%)	
Positive one years earlier			
No	739 (93.4%)	16 (61.5%)	0.05
Yes	52 (6.6%)	10 (38.5%)	
Suspension of the healthy status			
No	747 (94.4%)	25 (96.2%)	1
Yes	44 (5.6%)	1 (3.8%)	
No. of cattle on the farm			
Mean (SD)	5.89 (29.6)	1.50 (3.20)	0.502
Median (Min, Max)	0 (0, 641)	0 (0, 11.0)	
No. of movements			
Mean (SD)	0.422 (1.11)	0.308 (1.19)	0.21
Median (Min, Max)	0 (0, 13.0)	0 (0, 6.0)	
No. of animals moved			
Mean (SD)	12.5 (90.4)	10 (36.5)	0.263
Median (Min, Max)	0 (0, 2000)	0 (0, 170)	
No. of heads on the farm			
Mean (SD)	236 (234)	260 (219)	0.461
Median (Min, Max)	179 (1, 3010)	176 (10, 905)	
Density of animals per km^2^			
Mean (SD)	552 (490)	634 (474)	0.278
Median (Min, Max)	444 (0, 2260)	513 (88.5, 1740)	
Density of farms per km^2^			
Mean (SD)	1.47 (1.01)	2.17 (0.795)	<0.05
Median (Min, Max)	1.17 (0.0767, 3.48)	2.48 (0.476, 3.07)	

**Table 2:** 2016 Fisher test and Wilcoxon test.

	Negative	Positive	*p*-Value
	(N = 753)	(N = 35)	
Presence of cattle on the farm			
No	436 (57.9%)	19 (54.3%)	0.804
Yes	317 (42.1%)	16 (45.7%)	
Presence of sheep and goats on the farm			
No	734 (97.5%)	34 (87.1%)	1
Yes	19 (2.5%)	1 (2.9%)	
Movements			
No	561 (74.5%)	26 (74.3%)	1
Yes	192 (25.5%)	9 (25.7%)	
Abortions			
No	732 (97.2%)	32 (91.4%)	0.149
Yes	21 (2.8%)	3 (8.6%)	
Positive three years earlier			
No	727 (96.5%)	31 (88.6%)	0.0502
Yes	21(2.8%)	3 (8.6%)	
Positive one years earlier			
No	738 (98.0%)	27 (77.1%)	<0.05
Yes	15 (2.0%)	8 (22.9%)	
Suspension of the healthy status			
No	711 (94.4%)	26 (74.3%)	<0.05
Yes	42 (5.6%)	9 (25.7%)	
No. of cattle on the farm			
Mean (SD)	5.78 (22.8)	3.71 (10.2)	0.944
Median (Min, Max)	0 (0, 399)	0 (0, 57.0)	
No. of movements			
Mean (SD)	0.541 (1.50)	0.514 (1.01)	0.888
Median (Min, Max)	0 (0, 23.0)	0 (0, 4.0)	
No. of animals moved			
Mean (SD)	11.9 (47.5)	10.5 (29.4)	0.914
Median (Min, Max)	0 (0, 496)	0 (0, 134)	
No. of heads on the farm			
Mean (SD)	249 (249)	273 (201)	0.172
Median (Min, Max)	191 (1, 3250)	202 (8.00, 1050)	
Density of animals per km^2^			
Mean (SD)	583 (521)	463 (274)	0.592
Median (Min, Max)	474 (0, 2800)	439 (7.54, 1370)	
Density of farms per km^2^			
Mean (SD)	1.43 (0.970)	2.01 (0.782)	<0.05
Median (Min, Max)	1.16 (0.0762, 3.35)	2.18 (0.342, 3.19)	

**Table 3:** 2017 Fisher test and Wilcoxon test.

	Negative	Positive	*p*-Value
	(N = 726)	(N = 40)	
Presence of cattle on the farm			
No	426(58.3%)	23 (57.5%)	1
Yes	300 (41.3%)	17 (42.5%)	
Presence of sheep and goats on the farm			
No	704 (97.0%)	40 (100%)	0.528
Yes	22 (3.0%)	0 (0%)	
Movements			
No	563 (77.5%)	26 (65%)	0.101
Yes	163 (22.5%)	14 (35%)	
Abortions			
No	699 (96.3%)	36 (90.0%)	0.121
Yes	27 (3.7%)	4 (10%)	
Positive three years earlier			
No	700 (96.3%)	37 (92.5%)	0.402
Yes	26 (3.6%)	3 (7.5%)	
Positive one years earlier			
No	708 (97.5%)	30 (75.0%)	<0.05
Yes	26 (3.6%)	3 (7.5%)	
Suspension of the healthy status			
No	698 (96.1%)	36 (90.0%)	<0.05
Yes	28 (3.9%)	4 (10%)	
No. of cattle on the farm			
Mean (SD)	6.12 (22.2)	1.85 (4.33)	0.752
Median (Min, Max)	0 (0, 311)	0 (0, 23.0)	
No. of movements			
Mean (SD)	0.521 (1.68)	0.850 (1.75)	0.0597
Median (Min, Max)	0 (0, 26.0)	0 (0, 9.0)	
No. of animals moved			
Mean (SD)	8.60 (41.2)	13.1 (42.7)	0.0599
Median (Min, Max)	0 (0, 567)	0 (0, 242)	
No. of heads on the farm			
Mean (SD)	261 (256)	239 (167)	0.89
Median (Min, Max)	203 (1, 3080)	186 (29.0, 626)	
Density of animals per km^2^			
Mean (SD)	548 (435)	805 (545)	<0.05
Median (Min, Max)	453 (0, 1950)	729 (85.7, 1950)	
Density of farms per km^2^			
Mean (SD)	1.38 (0.943)	2.18 (0.793)	<0.05
Median (Min, Max)	1.17 (0.0757, 3.27)	2.31 (0.120, 3.27)	

**Table 4:** 2018 Fisher test and Wilcoxon test.

	Negative	Positive	*p*-Value
	(N = 696)	(N = 60)	
Presence of cattle on the farm			
No	417 (59.9%)	37 (61.7%)	0.898
Yes	279 (40.1%)	23 (38.3%)	
Presence of sheep and goats on the farm			
No	672 (96.6%)	59 (98.3%)	0.716
Yes	24 (3.4%)	1 (1.7%)	
Movements			
No	522 (75%)	53 (88.3%)	<0.05
Yes	174 (25%)	7 (11.7%)	
Abortions			
No	670 (96.3%)	58 (96.7%)	1
Yes	26 (3.7%)	4 (10%)	
Positive three years earlier			
No	678 (97.4%)	57 (95%)	0.495
Yes	18 (2.6%)	3 (5%)	
Positive one years earlier			
No	679 (97.6%)	41 (68.3%)	<0.05
Yes	17 (2.4%)	19 (31.7%)	
Suspension of the healthy status			
No	670 (97.6%)	57 (95.0%)	0.889
Yes	26 (3.7%)	3 (5%)	
No. of cattle on the farm			
Mean (SD)	5.20 (20.5)	16 (89.1)	0.575
Median (Min, Max)	0 (0, 292)	0 (0, 661)	
No. of movements			
Mean (SD)	0.506 (1.51)	0.267 (1.01)	<0.05
Median (Min, Max)	0 (0, 25.0)	0 (0, 7.0)	
No. of animals moved			
Mean (SD)	11.2 (44.6)	3.83 (13.6)	<0.05
Median (Min, Max)	0 (0, 433)	0 (0, 70)	
No. of heads on the farm			
Mean (SD)	262 (262)	264 (202)	0.384
Median (Min, Max)	204 (1, 3060)	253 (13, 626)	
Density of animals per km^2^			
Mean (SD)	537 (454)	738 (433)	<0.05
Median (Min, Max)	429 (0, 2540)	650 (75.9, 1750)	
Density of farms per km^2^			
Mean (SD)	1.32 (0.919)	2.25 (0.679)	<0.05
Median (Min, Max)	1.09 (0.07771, 3.15)	2.45 (0.0911, 3.12)	

**Table 5:** 2019 Fisher test and Wilcoxon test.

	Negative	Positive	*p*-Value
	(N = 653)	(N = 87)	
Presence of cattle on the farm			
No	411 (62.9%)	64 (73.6%)	0.0684
Yes	242 (37.1%)	23 (26.4%)	
Presence of sheep and goats on the farm			
No	626 (95.9%)	87 (100%)	0.104
Yes	27 (4.1%)	0 (0%)	
Movements			
No	536 (82.1%)	71 (81.6%)	1
Yes	117 (17.9%)	16 (18.4%)	
Abortions			
No	627 (96.0%)	79 (90.8%)	0.0562
Yes	26 (4%)	8 (9.2%)	
Positive three years earlier			
No	628 (96.2%)	82 (94.3%)	0.573
Yes	25 (3.8%)	5 (5.7%)	
Positive one years earlier			
No	626 (95.9%)	60 (69.0%)	<0.05
Yes	27 (4.1%)	27 (31.0%)	
Suspension of the healthy status			
No	638 (97.7%)	79 (90.8%)	<0.05
Yes	15 (2.3%)	8 (9.2%)	
No. of cattle on the farm			
Mean (SD)	4.99(18.1)	2.66 (16.1)	<0.05
Median (Min, Max)	0 (0, 254)	0 (0, 146)	
No. of movements			
Mean (SD)	0.415 (1.41)	0.402 (1.08)	0.883
Median (Min, Max)	0 (0, 20.0)	0 (0, 6.0)	
No. of animals moved			
Mean (SD)	9.32 (46.3)	15.8 (68.6)	0.807
Median (Min, Max)	0 (0, 584)	0 (0, 544)	
No. of heads on the farm			
Mean (SD)	269 (272)	268 (195)	0.277
Median (Min, Max)	205 (1, 3030)	245 (10, 1020)	
Density of animals per km^2^			
Mean (SD)	507 (418)	684 (366)	<0.05
Median (Min, Max)	420 (0, 2420)	738 (0, 1860)	
Density of farms per km^2^			
Mean (SD)	1.24 (0.879)	2.13 (0.708)	<0.05
Median (Min, Max)	1.01 (0.0796, 3.09)	2.34 (0.233, 3.04)	

**Table 6:** 2020 Fisher test and Wilcoxon test.

	Negative	Positive	*p*-Value
	(N = 611)	(N = 105)	
Presence of cattle on the farm			
No	384 (62.8%)	82 (78.1%)	<0.05
Yes	227 (37.2%)	23 (21.9%)	
Presence of sheep and goats on the farm			
No	590 (96.6%)	104 (99.0%)	0.291
Yes	21 (3.4%)	1 (1.0%)	
Movements			
No	487 (79.7%)	93 (88.6%)	<0.05
Yes	124 (20.3%)	12 (11.4%)	
Abortions			
No	591 (96.7%)	96 (91.4%)	<0.05
Yes	124 (20.3%)	9 (8.6%)	
Positive three years earlier			
No	581 (95.1%)	99 (94.3%)	0.915
Yes	30 (4.9%)	6 (5.7%)	
Positive one years earlier			
No	582 (95.3%)	64 (61.0%)	<0.05
Yes	29 (4.7%)	41 (39.0%)	
Suspension of the healthy status			
No	601 (98.4%)	88 (83.8%)	<0.05
Yes	10 (1.6%)	17 (16.2%)	
No. of cattle on the farm			
Mean (SD)	6.12 (30.4)	3.10 (13.5)	<0.05
Median (Min, Max)	0 (0, 590)	0 (0, 108)	
No. of movements			
Mean (SD)	0.656 (2.13)	0.190 (0.773)	<0.05
Median (Min, Max)	0 (0, 23.0)	0 (0, 7.0)	
No. of animals moved			
Mean (SD)	13.2 (73.2)	5.55 (26.5)	<0.05
Median (Min, Max)	0 (0, 1240)	0 (0, 233)	
No. of heads on the farm			
Mean (SD)	276 (281)	255 (246)	0.4
Mediana (Min, Max)	209 (1, 3010)	173 (11, 1380)	
Density of animals per km^2^			
Mean (SD)	467 (386)	613 (402)	<0.05
Median (Min, Max)	382 (0, 2060)	500 (25.9, 1820)	
Density of farms per km^2^			
Mean (SD)	1.18 (0.853)	1.97 (0.740)	<0.05
Median (Min, Max)	0.975 (0.0786, 3.08)	2.07 (0.304, 3.08)	

**Table 7:** OR = Odds Ratio, CI = Confidence Interval, * = iteration between the one variable.

Characteristic	OR	95% CI	*p*-Value
years * movements			
2015 * 1	1.012	1.003, 1.044	>0.05
2016 * 1	1.043	1.019, 1.097	<0.05
2017 * 1	1.085	1.041, 1.169	<0.05
2018 * 1	1.038	1.015, 1.093	<0.05
2019 * 1	1.152	1.075, 1.294	<0.05
2020 * 1	1.102	1.046, 1.214	<0.05
Health status suspension	3.510	2.165, 5.691	<0.05
Density of farms per km^2^	3.132	2.583, 3.798	<0.05
Abortions	2.442	1.395, 4.276	<0.05

**Table 8:** OR = Odds Ratio, CI = Confidence Interval.

Characteristic	OR	95% CI	*p*-Value
Intercept	0.007	0.004, 0.013	<0.05
2016	1.481	0.854, 2.570	<0.05
2017	1.854	1.081, 3.179	<0.05
2018	3.200	1.921, 5.333	<0.05
2019	5.517	3.365, 9.045	<0.05
2020	7.927	4.842, 12.978	<0.05
pre-2015 positivity	3.510	2.165, 5.691	<0.05

## Data Availability

Data is not available due to privacy.
